# Strigolactones Interact With Nitric Oxide in Regulating Root System Architecture of *Arabidopsis thaliana*

**DOI:** 10.3389/fpls.2020.01019

**Published:** 2020-07-03

**Authors:** Dóra Oláh, Gábor Feigl, Árpád Molnár, Attila Ördög, Zsuzsanna Kolbert

**Affiliations:** Department of Plant Biology, University of Szeged, Szeged, Hungary

**Keywords:** *Arabidopsis thaliana*, nitric oxide, root, *S*-nitrosoglutathione reductase, strigolactone

## Abstract

Both nitric oxide (NO) and strigolactone (SL) are growth regulating signal components in plants; however, regarding their possible interplay our knowledge is limited. Therefore, this study aims to provide new evidence for the signal interplay between NO and SL in the formation of root system architecture using complementary pharmacological and molecular biological approaches in the model *Arabidopsis thaliana* grown under stress-free conditions. Deficiency of SL synthesis or signaling (*max1-1* and *max2-1*) resulted in elevated NO and *S*-nitrosothiol (SNO) levels due to decreased *S*-nitrosoglutathione (GSNO) reductase (GSNOR) protein abundance and activity indicating that there is a signal interaction between SLs and GSNOR-regulated levels of NO/SNO. This was further supported by the down-regulation of SL biosynthetic genes (*CCD7, CCD8* and *MAX1*) in GSNOR-deficient *gsnor1-3*. Based on the more pronounced sensitivity of *gsnor1-3* to exogenous SL (*rac-*GR24, 2 µM), we suspected that functional GSNOR is needed to control NO/SNO levels during SL-induced primary root (PR) elongation. Additionally, SLs may be involved in GSNO-regulated PR shortening as suggested by the relative insensitivity of *max1-1* and *max2-1* mutants to exogenous GSNO (250 µM). Collectively, our results indicate a connection between SL and GSNOR-regulated NO/SNO signals in roots of *A. thaliana* grown in stress-free environment. As this work used *max2-1* mutant and *rac*-GR24 exerting unspecific effects to both SL and karrikin signaling, it cannot be ruled out that karrikins are partly responsible for the observed effects, and this issue needs further clarification in the future.

## Introduction

Strigolactones (SLs) have been first identified as germination inducers of parasite plants in the 1960s (Cook et al., 1966) and since then, they have been found to be phytohormones due to their multiple roles in regulating growth and developmental processes of higher plants (Gomez-Roldan et al., 2008; Umehara et al., 2008; Zwanenburg and Blanco-Ania, 2018; Bouwmeester et al., 2019).

SLs as terpenoid lactones can be categorized as canonical SLs containing ABC ring and noncanonical SLs lacking such a ring (Al-Babili and Bouwmeester, 2015; Waters et al., 2017). SLs are synthetized from carotenoids in the plastids with the involvement of enzymes such as beta-carotene-isomerase (D27), two carotenoid cleavage dioxygenases (CCD7/MAX3 and CCD8/MAX4), cytochrome P450 (MAX1), and LATERAL BRANCHING OXIDOREDUCTASE (Alder et al., 2012; Brewer et al., 2016). Following its transport into the cytoplasm, carlactone is converted into carlactonoic acid which is the common precursor of the naturally occurring SLs (Jia et al., 2019). Recently, the direct conversion of carlactonoic acid to orobanchol without passing through 4-deoxyorobanchol has been described (Wakabayashi et al., 2019). Moreover, a cytochrome P450 and a 2-oxoglutarate-dependent dioxygenase genes were identified being involved in SL synthesis in *Lotus japonicus* (Mori et al., 2020), and hydroxyl carlactone derivatives as relevant intermediaries in SL synthesis have been identified in *Arabidopsis* (Yoneyama et al., 2020). Despite the active research, our knowledge about the details of SL biosynthesis after carlactone is still limited (Bouwmeester et al., 2019). It has been shown that SLs are synthetized in both the root and the shoot and that the SL signal can spread from the root to the shoot system (Foo et al., 2001).

The perception of SLs involves the SL receptor DWARF14 (D14) protein having α/β fold hydrolase activity. The intact SL molecule promotes D14 activation which in turn deactivates bioactive SLs by the hydrolytic degradation following signal transmission (Seto et al., 2019). Consequently, the activated D14 can bind the MORE AXILLARY GROWTH2 (MAX2/D3) F-box type protein which assigns DWARF53 and SMXLs repressors for proteasomal degradation resulting in the induction of gene expression (Shabek et al., 2018; Bouwmeester et al., 2019). Recently, MAX2 was implicated as a regulator of karrikin (KAR) signaling (Nelson et al., 2011), and SMXL/D53, the downstream targets of MAX2 are responsible for the discrimination of SL and KAR signal pathways (Soundappan et al., 2015). The interference between SL and KAR signaling is further supported by the fact that *rac*-GR24 (racemic mixtures of GR24 stereoisomers) activates both signal pathways, thus exerts also non-SL-specific effects (Scaffidi et al., 2014; Li et al., 2016). The SL-induced gene expression manifests in physiological effects such as the inhibition of shoot branching, shaping of root system architecture, inducing leaf senescence (Pandey et al., 2016; Waters et al., 2017; Marzec and Melzer, 2018). Recently, Villaécija-Aguilar and co-workers (2019) added that root traits like root hair development, root skewing, straightness, and diameter are regulated by KAR signaling, while both KAR and SL pathways contribute to the regulation of lateral root density and epidermal cell length. Furthermore, SLs have been implicated in plant stress responses to diverse abiotic factors (reviewed by Mostofa et al., 2018) like nutrient deficiency (Kohlen et al., 2011), salinity and drought (Ha et al., 2014; Wang et al., 2019, reviewed by Mostofa et al., 2018) or chilling (Cooper et al., 2018).

Similar to SLs, research over the past 40 years has revealed that the gaseous signal molecule nitric oxide (NO) is a multifunctional growth regulator in plants (Kolbert et al., 2019a). While, the ability of SL synthesis is a unique feature of plants (Walker et al., 2019), any living organism is capable of the synthesis of NO. Algae utilize NO synthase (NOS)-like enzyme system for producing NO (Foresi et al., 2010; Foresi et al., 2015; Weisslocker-Schaetzel et al., 2017) while in higher land plants NOS gene homolog to animal gene has not been found (Jeandroz et al., 2016; Santolini et al., 2017; Hancock and Neill, 2019). The ability of NO liberation via NOS-system may be lost during the evolution of land plants (Fröhlich and Durner, 2011), which takes up high amounts of nitrate, and their physiological functions are greatly determined by nitrate acquisitions. A key process in nitrate-dependent NO synthesis of plants indirectly involves nitrate reductase (NR) activity which transfers electron from NAD(P)H to the NO-forming nitrite reductase (NOFNiR). This enzyme catalyzes the reduction of nitrite to NO (Chamizo-Ampudia et al., 2016; Chamizo-Ampudia et al., 2017). NO is synthetized endogenously within the plant body in a wide variety of tissues, and NO can also be taken up from the atmosphere or from the soil (Cohen et al., 2009). In biological systems, NO reacts with glutathione to form *S*-nitrosoglutathione (GSNO) being a less reactive and more stable molecule than NO. GSNO is able to release NO and can achieve long distance movement of NO signal *via* the xylem (Durner et al., 1999; Díaz et al., 2003; Barroso et al., 2006). Intracellular levels of GSNO are controlled by the activity of GSNO reductase (GSNOR) enzyme (Feechan et al., 2005; Lee et al., 2008; Chen et al., 2009) catalyzing the conversion of GSNO to GSSG and NH_3_ in the presence of NADH (Jahnová et al., 2019).

Unlike SLs, the signal of NO isn't perceived by specific receptor, but the transfer of NO bioactivity is achieved by direct modification of target proteins. Cysteine *S*-nitrosation, tyrosine nitration, and metal nitrosylation are three major NO-dependent posttranslational modifications being physiologically relevant (Astier and Lindermayr, 2012). Additionally, the link between NO-related signaling and Ca^2+^-, cGMP-, MAPK-, and PA-dependent signaling has also been revealed in diverse physiological processes (Pagnussat et al., 2004; Lanteri et al., 2008; Astier et al., 2011; Jiao et al., 2018). Like SLs, NO affects a range of physiological traits including seed development, vegetative and generative development like pollen tube growth, seed germination, root growth, gravitropism, flowering, fruit ripening (reviewed in Kolbert and Feigl, 2017). Additionally, NO also participates in responses of plants to abiotic stresses like salinity, drought, heavy metal, low oxygen availability, or temperature stresses (Fancy et al., 2017).

Based on the stimulating effect of NO on plant germination, vegetative growth or fruit ripening, NO-releasing substances such as nanoparticles could be effectively applied in agricultural practice (Rodríguez-Ruiz et al., 2019). Similarly, SLs and their agonists and antagonists may have a great potential for agricultural applications. Beyond plant protection, SLs may be used to improve the architecture of crops as well (Vurro et al., 2016; Takahashi and Asami, 2018).

It is sure that both NO and SL are important growth regulating signals of practical significance in plants. However, their interplay has been poorly examined. The majority of the few articles dealing with SL–NO interplay focus on the root system of crops like sunflower (Barthi and Bhatla, 2015), maize (Manoli et al., 2016), and rice (Sun et al., 2014) grown in the presence of different nutrient supplies. Collectively, these studies revealed that NO is an upstream regulator of SL signaling; however, the nature of the NO–SL relationship depends on the nutrient availability. During nitrate‐induced root elongation, NO reduces SL biosynthesis thus resulting in alterations of PIN‐mediated auxin transport leading to cell elongation. Exogenous SL induces NO production suggesting negative feedback regulation of SL levels (Manoli et al., 2016). Low N and P availability triggers NO formation which in turn induces the proteasomal‐degradation of D53 repressor protein and consequently intensifies SL signaling leading to root elongation (Sun et al., 2016). To clarify the role of SLs in root development, Marzec and Melzer (2018) recommended to perform experiments with plants grown during stress-free conditions. Because of the above reasons, this study aims to provide new evidence for the signal interplay between NO and SL in the formation of root system architecture using complementary pharmacological and molecular biological approaches in the model *Arabidopsis thaliana* grown under stress-free conditions.

## Materials and Methods

### Plant Material and Growth Conditions

Seeds of *Arabidopsis thaliana* wild-type (WT, Col-0), and their mutant lines *gsnor1-3* (Chen et al., 2009), *35S:FLAG-GSNOR1* (Frungillo et al., 2014), *max1-1*, *max2-1* (Stirnberg et al., 2002) were surfaced sterilized with 70% (v/v) ethanol for 1 min and with 30% sodium hypochlorite solution (1:3) for 15 min then washed five times with sterile distilled water. Seeds (approx. 30 seeds/Petri dish) were then transferred to half strength Murashige and Skoog medium (1% sucrose, 0.8% agar). Petri dishes were kept in a greenhouse under controlled conditions (photon flux density of 150 µmol m^−2^ s^−1^, 12/12 h light and dark cycle, relative humidity of 55–60%, temperature of 25 ± 2°C) for 7 days.

### Treatments

Stock solution of *rac*-GR24 and TIS108 (both purchased from Chiralix B.V., Nijmegen, Netherlands) was prepared in acetone or in DMSO, respectively. Appropriate volumes of stock solutions were added to the medium following sterilization through sterile syringe yielding 2 µM GR24 or 5 µM TIS108 concentrations in the media. These concentrations were chosen in pilot experiments using several doses (1, 2, 5 µM for GR24 and 1, 5, 10 µM for TIS108). Stock solutions of GSNO and 2-(4-carboxyphenyl)-4,4,5,5-tetramethylimidazoline-1-oxyl-3-oxide (cPTIO) were prepared in DMSO and were diluted to the final concentrations (250 µM GSNO and 800 µM cPTIO) with distilled water. Four days after placing the seeds on the media, GSNO and cPTIO solutions were added to the surface of the agar containing the root system. One milliliter of GSNO or cPTIO was added per Petri dish using 2-ml syringe and sterile filter.

### Morphological Measurements

Primary root (PR) lengths of *Arabidopsis* seedlings were measured and expressed in mm. Lateral roots within the primary root (smaller than stage VII) were considered as lateral root primordia (LR_prim_), whereas visible laterals which have already grown outside the PR were considered as emerged LRs (LR_em_, larger than stage VII, Malamy and Benfey, 1997; Feigl et al., 2019). The number of LR_prim_ and LR_em_ was determined by using Zeiss Axiovert 200 inverted microscope and 20× objective (Carl Zeiss, Jena, Germany). LR density (number mm^−1^) was calculated by dividing total number of LRs with PR length. The experiments were performed three times with 20 samples each (*n* = 60).

### Detection of NO Levels

Levels of NO were detected with the fluorophore, 4-amino-5-methylamino-2′-7′-difluorofluorescein diacetate (DAF-FM DA). *Arabidopsis* seedlings were incubated in 10 µM dye solution for 30 min, in darkness, at room temperature and washed two times with TRIS-HCl buffer (10 mM, pH 7.4) according to Kolbert et al. (2012). Stained root samples were observed under Axiovert 200M (Carl Zeiss, Jena, Germany) fluorescent microscope equipped with digital camera (Axiocam HR) and filter set 10 (excitation 450–490 nm, emission 515–565 nm) Fluorescence intensities in the PRs were measured on digital images using Axiovision Rel. 4.8 software within circles of 38 µm radii. This analysis was carried out three times with 10 root tips examined (*n* = 10).

### Determination of *S*-nitrosothiol Contents

The amount of SNO was quantified by Sievers 280i NO analyser (GE Analytical Instruments, Boulder, CO, USA) according to Kolbert et al. (2019b). Briefly, 250 mg of *Arabidopsis* seedlings was mixed with double volume of 1× PBS buffer (containing 10 mM N-ethylmaleimide and 2.5 mM EDTA, pH 7.4) and were grounded using Fast Prep ^®^ Instrument (Savant Instruments Inc., Holbrook, NY). Samples were centrifuged twice for 15 min (20,000 g, 4°C). The supernatants were incubated with 20 mM sulphanilamide. 250 µl of the samples was injected into the reaction vessel filled with potassium iodide. SNO concentrations were quantified with the help of NO analysis software (v3.2). Measurement of SNO levels was performed on three separate plant generations with five technical replicates in each (*n* = 5).

### Western Blot Analysis of GSNOR Protein Abundance

Whole *Arabidopsis* seedlings were grounded with extraction buffer (50 mM TRIS-HCl, pH 7.6–7.8) and centrifuged (4°C, 9300 g, 20 min). Protein extract was treated with 1% proteinase inhibitor and stored at −80°C. Protein concentrations were determined using the Bradford (1976) assay.

Fifteen microliters of denaturated protein extract was subjected to SDS-PAGE on 12% acrylamide gel. Proteins were transferred to PVDF membranes using the wet blotting procedure (25 mA, 16 h). After that, membranes were used for cross-activity assays with rabbit polyclonal antibody against GSNOR (1:2,000). Immunodetection was performed by using affinity, isolated goat anti-rabbit IgG-alkaline phosphatase secondary antibody at a dilution of 1:10,000, and bands were visualized by using the NBT/BCIP reaction. Protein bands were quantified by Gelquant software (provided by biochemlabsolutions.com). Western blot was carried out on three separate protein extracts from independent plant generations, at least two times per extract.

### Spectrophotometric Measurement of GSNOR Activity

The specific activity of GSNOR was measured by monitoring the NADH oxidation in the presence of GSNO at 340 nm (Sakamoto et al., 2002). Plant homogenate was centrifuged (14,000 g, 20 min, 4°C), and 100 µg of protein extract was incubated in 1 ml reaction buffer (20 mM Tris-HCl pH 8.0, 0.5 mM EDTA, 0.2 mM NADH). Data are expressed as nmol NADH min^−1^ mg protein^−1^. This measurement was performed on three separate plant generations with five technical replicates in each (*n* = 5).

### Quantitative Real Time PCR Analysis

The expression rates of *Arabidopsis* genes *(NIA1, NIA2, GLB1, GLB2, GSNOR1, CCD7, CCD8, D14, MAX1, MAX2)* were determined by quantitative real-time reverse transcription PCR (RT-qPCR). RNA was purified from 90 mg of 7-day-old seedlings by using a NucleoSpin RNA Plant mini spin kit (Macherey-Nagel) according to the manufacturer's instruction. Furthermore, an additional DNAase digestion and purifying step was applied (ZYMO Research), and cDNA was synthetized using RevertAid reverse transcriptase. Primer3 software was used for designing primers. The primers used for RT-qPCR analyses are listed in **Table S1**. The expression rates of the NO- and SL associated genes were detected by quantitative real time PCR machine (qTOWER 2.0, Jena Instruments) using SYBR Green PCR Master Mix (Thermo Mix) (Gallé et al., 2009). Data were analyzed by using qPCRsoft3.2 software (Jena Instruments). Data were normalized to the transcript levels of the control samples; *ACTIN2* (At3918780) and *GAPDH2* (At1913440) were used as internal controls (Papdi et al., 2008). Each reaction was carried out in three replicates using cDNA synthesized from independently extracted RNAs. These analyses were performed on three separate plant generations with three technical replicates in each (*n* = 3).

### Measurement of NO Liberation Capacity of GSNO

NO-sensitive electrode (ISO-NOP 2 mm, World Precision Instrument) was calibrated using a method of Zhang (2004). Donor solution (1 ml 250 µM GSNO in distilled water) was prepared and placed under illumination (150 µmol m^−2^ s^−1^) in the greenhouse in order to stimulate conditions similar to treatment conditions. To ensure constant mixing of the solution magnetic stirrer was applied during the measurement. NO concentration (nM) was calculated from a standard curve. The standard curve and the results are presented in **Figure S2**. This measurement was carried out three times with three technical replicates in each (*n* = 3).

### Statistical Analysis

All results are expressed as mean ± SE. Graphs were prepared in Microsoft Excel 2010 and in SigmaPlot 12. For statistical analysis, Duncan's multiple range test (one-way ANOVA, P ≤ 0.05) was used in SigmaPlot 12. For the assumptions of ANOVA, we used Hartley's F_max_ test for homogeneity and the Shapiro–Wilk normality test.

## Results and Discussion

### Root System of GSNOR- and SL Mutant *Arabidopsis* Seedlings

**Figure 1:**
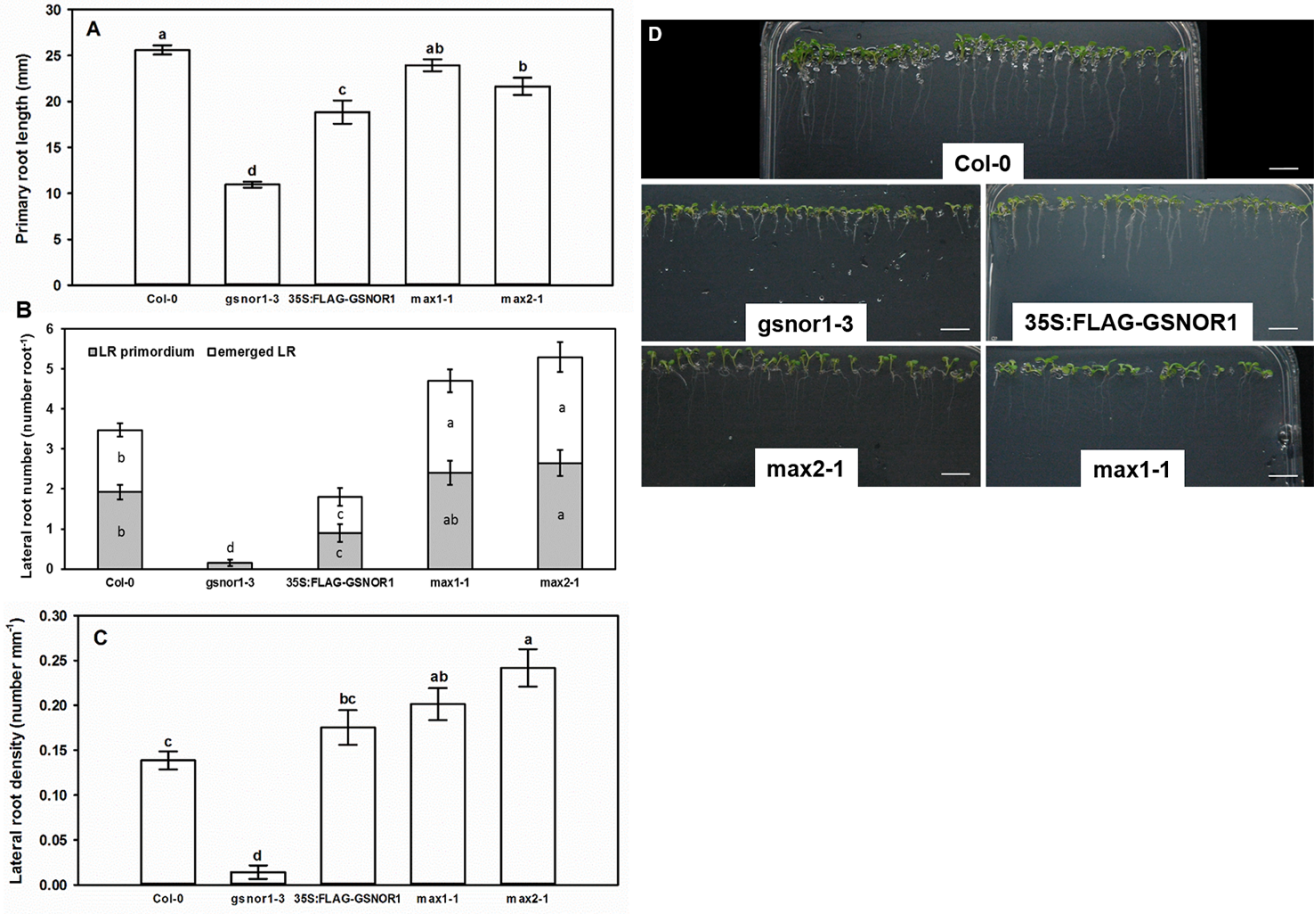
Primary root length (mm, **A**), lateral root number (number root^−1^, **B**) and lateral root density (number mm^−1^, **C**) in 7-day-old Col-0, GSNOR- and SL mutant *Arabidopsis* lines grown during stress-free conditions. Different letters indicate significant differences according to Duncan's test (*n =* 60, P ≤ 0.05). **(D)** Representative photographs taken from 7-day-old *Arabidopsis* seedlings of different mutant lines grown on ½ MS medium. Bars = 1 cm.

Compared to the wild-type (Col-0), the PR of *gsnor1-3* mutant was by 57% shorter; its root system contained very few LRs, and consequently its LR density was low (**Figure 1**) indicating that GSNOR activity is necessary for normal root development (Lee et al., 2008; Holzmeister et al., 2011; Kwon et al., 2012; Shi et al., 2015). Similarly, *35S:FLAG-GSNOR1* seedlings had shortened PRs and reduced numbers of laterals resulting in WT-like LR density, and the LR primordia to emerged LR ratio was similar to that of Col-0. As for the *max1-1* mutant, WT-like PR length was accompanied by increased number of emerged LRs and by consequently enhanced LR density compared to Col-0. The PR of *max2-1* mutant proved to be slightly (by 14%) shorter than in Col-0 and the LR number was significantly increased. The branched root systems of *max1-1* and *max2-1* suggest that MAX1-dependent SL biosynthesis and MAX2-associated SL-signaling inhibit LR development as was published previously by others (Kapulnik et al., 2011; Ruyter-Spira et al., 2011). The LR_prim_ : LR_em_ ratio was similar in Col-0 and the mutants suggesting that SLs similarly influence both the initiation and the emergence of LRs. However, *max2-1* mutant has been proven to transmit both SL and KAR signals, thus the involvement of KAR in shaping root system architecture cannot be ruled out using this mutant (Villaécija-Aguilar et al., 2019).

### Levels of NO and SNO in GSNOR- and SL Mutant *Arabidopsis* Seedlings

**Figure 2:**
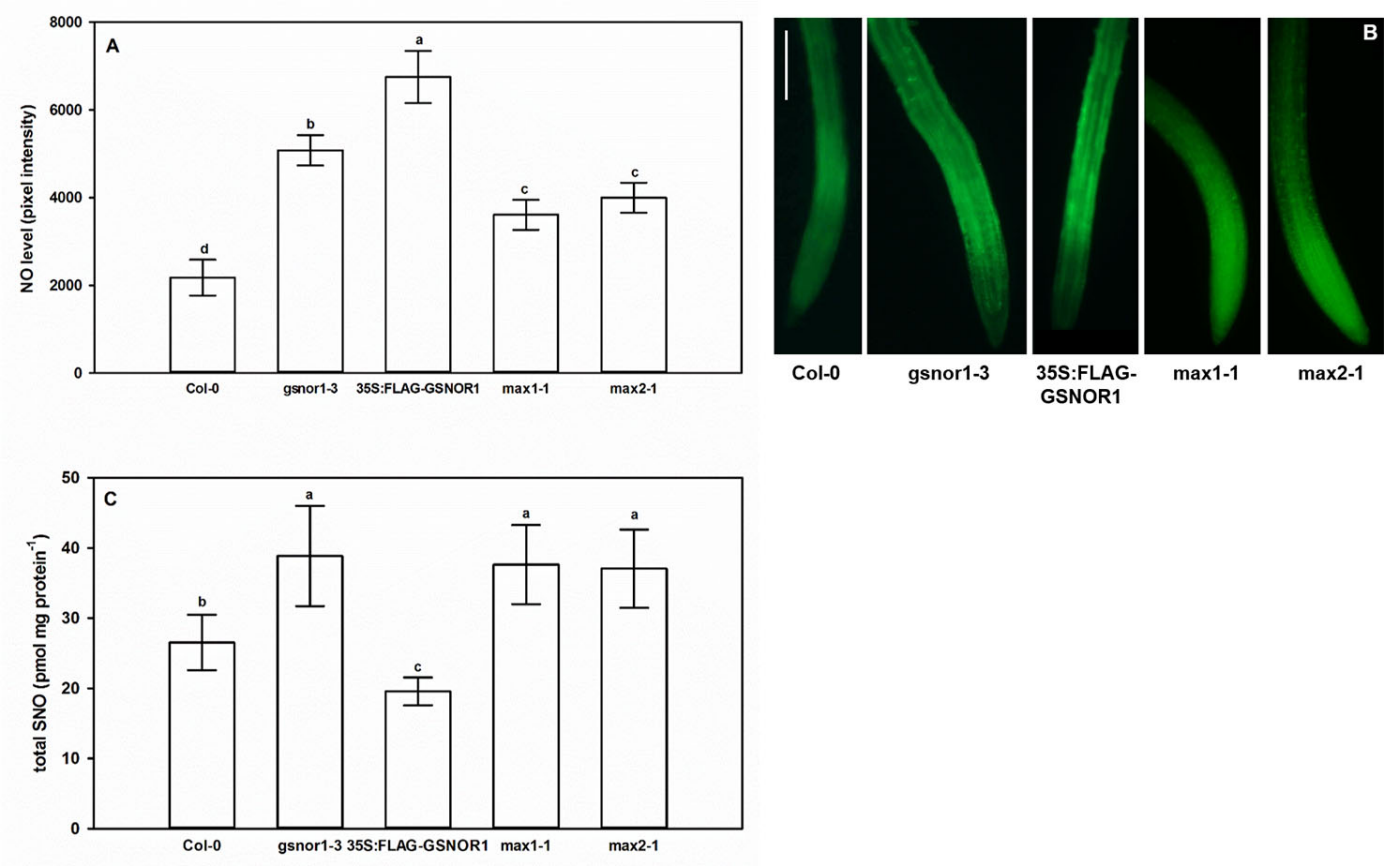
Nitric oxide levels (pixel intensity, **A**) and SNO levels (pmol mg protein^−1^, **C**) in Col-0, GSNOR- and SL mutant *Arabidopsis* seedlings grown during stress-free conditions for 7 days. Different letters indicate significant differences according to Duncan's test (*n =* 10 or 5, P ≤ 0.05). **(B)** Representative microscopic images showing DAF-FM DA-stained root tips of examined *Arabidopsis* lines. Bar = 100 µm.

As shown in **Figure 2**, the level of NO and SNO in *gsnor1-3* was higher than in Col-0, while in *35S:FLAG-GSNOR1* plants, the increased endogenous NO level was accompanied by lower SNO levels than in the WT. The origin of the high NO level in the mutants is different. In *35S:FLAG-GSNOR1*, elevated nitrate content and nitrate reductase activity were observed which may result in the enhanced NO level (Frungillo et al., 2014), while in *gsnor1-3* the lack of GSNOR1 leads to enhanced SNO and consequently high NO contents. Based on these, applying *35S:FLAG-GSNOR1* mutant allows to draw conclusions about nitrate-derived NO while with the help of *gsnor1-3* mutant we can get information about the role of GSNOR-dependent NO removal. Moreover, the similar root system of the GSNOR mutants (**Figure 1**) can be explained by their high NO contents which are known to reduce auxin maximum and consequently cause PR shortening (Fernández-Marcos et al., 2011; Shi et al., 2015). In *max1-1* and *max2-1* significantly increased NO level and SNO content were detected compared to Col-0 (**Figure 2**).

**Figure 3:**
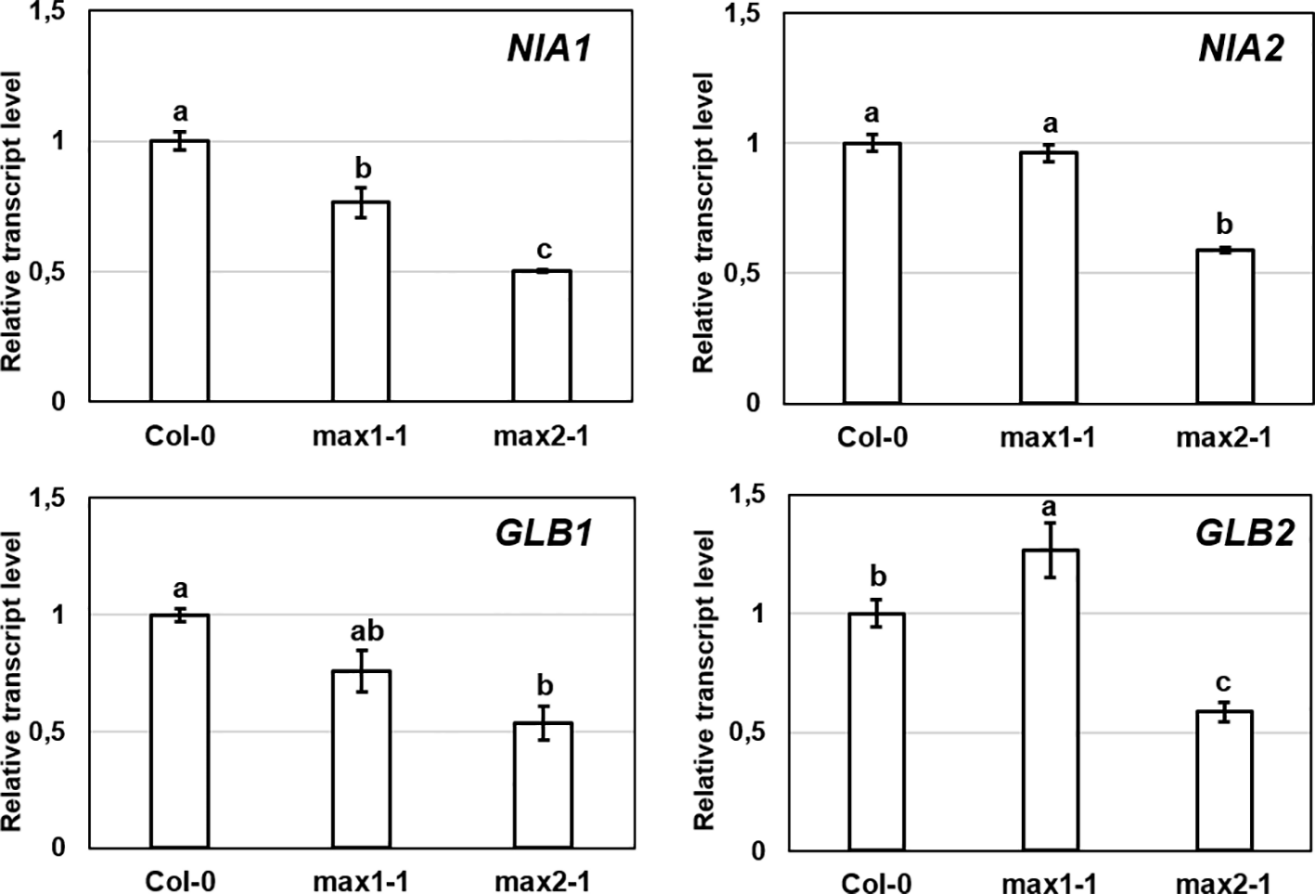
Relative transcript level of selected NO-associated genes (*NIA1, NIA2, GLB1, GLB2*) in control Col-0, *max1-1* and *max2-1 Arabidopsis* seedlings. Different letters indicate significant differences according to Duncan's test (*n =* 3, P ≤ 0.05). Data were normalized using the *A. thaliana ACTIN2* and *GAPDH2* genes as internal controls. The relative transcript level in Col-0 control samples was arbitrarily considered to be 1 for each gene.

Expressions of genes involved in NO metabolism (*NIA1, NIA2, GLB1, GLB2*) in *max1-1* mutants were similar to Col-0, but all examined genes were slightly down-regulated in *max2*-*1* (**Figure 3**). However, the changes were small and were not detectable in both *max* mutants, suggesting that these genes may not play a significant role in the regulation of NO in the absence of SLs.

**Figure 4:**
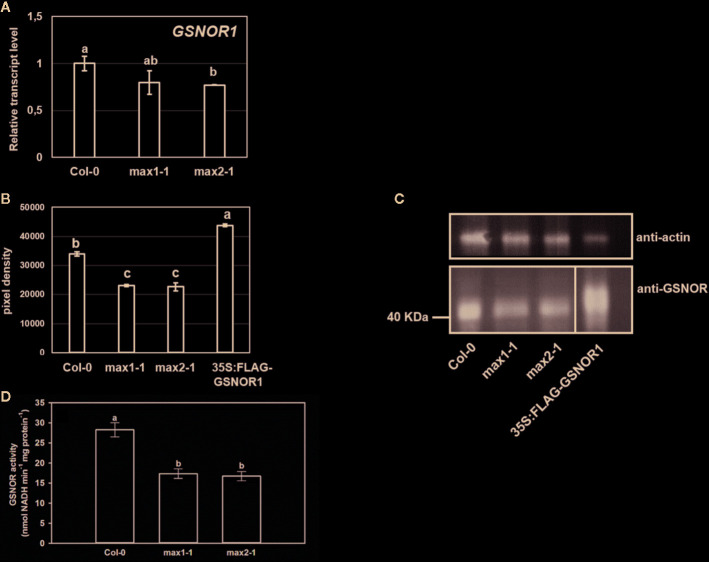
Relative transcript level **(A)** of *GSNOR1* in Col-0, *max1-1* and *max2-1* seedlings. **(B, C)** Protein abundance of GSNOR in *max* mutants and *35S:FLAG-GSNOR1* (as a positive control). Anti-actin was used as a loading control. **(D)** GSNOR activity (nmol NADH min^−1^ mg protein^−1^) in Col-0, *max1-1* and *max2-1* seedlings. Different letters indicate significant differences according to Duncan's test (*n =* 3 or 5, P ≤ 0.05).

Higher NO levels of the *max* mutants may be associated with higher SNO levels. GSNOR is a key regulator of SNO metabolism (Lindermayr, 2018), thus we assumed that *max* mutants show differences in association with GSNOR enzyme. Although, there were no relevant differences in the rates of *GSNOR1* expression in the plant lines (**Figure 4A**), the GSNOR protein abundance was significantly lower in *max* mutants compared to Col-0 (**Figures 4B, C**), and also the activity of the enzyme was decreased in *max1-1* and *max2-1* mutant seedlings (**Figure 4D**) which may provide the explanation for the elevated SNO and NO levels (**Figure 3**). These results indicate that SL (and/or possibly KAR) deficiency posttranscriptionally influence GSNOR enzyme resulting in decreased SNO/NO levels. As NO acts through SLs (and/or possibly KAR) to regulate root development, the effect of SL on GSNOR-regulated NO levels may be considered as compensatory feedback mechanism. Next, we examined the responses of GSNOR deficient and -overexpressing *Arabidopsis* lines to exogenous application of SL analog GR24 and SL synthesis inhibitor TIS108.

### The Effect of SL Analog and Inhibitor on Root System and NO-Associated Genes in *Arabidopsis*

**Figure 5:**
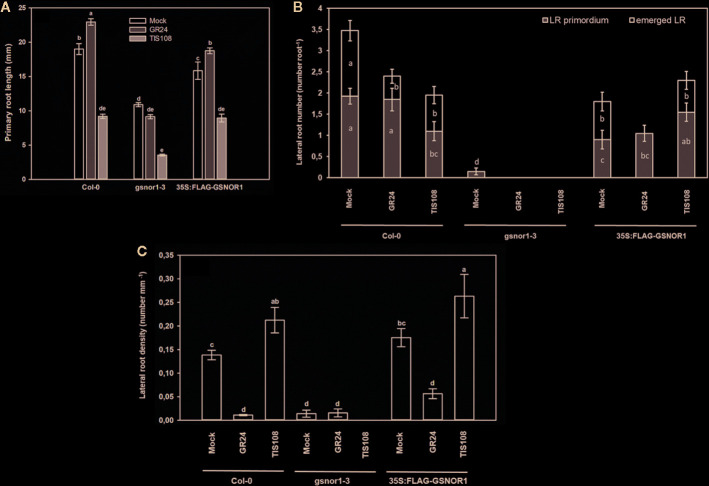
Primary root length (mm, **A**), lateral root number (number root^−1^, **B**) and lateral root density (number mm^−1^, **C**) in Col-0, *gsnor1-3* and *35S:FLAG-GSNOR1 Arabidopsis* seedlings grown in the absence (Mock) or in the presence of GR24 (2 µM) or TIS108 (5 µM). Different letters indicate significant differences according to Duncan's test (*n =* 60, P ≤ 0.05).

Similar to previously published results, GR24 treatment induced PR elongation in Col-0 *Arabidopsis* plants (Ruyter-Spira et al., 2011; Sun et al., 2014; Marzec, 2016), while TIS108 caused 50% inhibition of it (**Figure 5A**). To prove the SL-specific and non-toxic effect of TIS108 on *Arabidopsis* root, we applied GR24 together with TIS108 on Col-0 and we included *max1-1* mutant as a TIS108-resistant line (**Figure S1**). The *max1-1* mutant proved to be less sensitive to the root growth inhibiting effect of TIS108 compared to the wild-type (**Figure S1A**), and GR24 partly reversed the root shortening effect of TIS108 in Col-0 (**Figure S1B**). These indicate that the applied concentration of TIS108 is not toxic and exerts its biological effect through SLs. In case of *gsnor1-3*, SL analog did not trigger PR elongation and TIS108 reduced PR length by 67% compared to the control. These suggest that the root system of *gsnor1-3* is more sensitive to modifications of SL levels meaning that functional GSNOR enzyme is needed to control NO/SNO levels and to the positive effect of GR24 on PR elongation. Presumably, in case of GSNOR deficiency, NO/SNO levels are not properly regulated and high NO/SNO levels may cause PR shortening instead of elongation (Fernández-Marcos et al., 2011). The root elongation response of *35S:FLAG-GSNOR1* to SL analog or inhibitor did not differ from that of Col-0 indicating that overexpressing GSNOR enzyme or nitrate-derived NO has no effect on SL-induced elongation (**Figure 5A**). Treatment with GR24 resulted in reduced LR_em_ number and unchanged LR_prim_ number (**Figure 5B**) suggesting that SLs influence LR emergence but not LR initiation. In GSNOR overexpressing line, GR24-induced inhibition of LR emergence proved to be more pronounced than in Col-0. Additionally, in the stunted root system of *gsnor1-3*, the number of LR primordia was completely reduced by GR24. These results regarding the inhibitory effect of SL analog GR24 support previously published results (Kapulnik et al., 2011; Ruyter-Spira et al., 2011; Arite et al., 2012; De Cuyper et al., 2015; Marzec, 2016). However, without using different GR24 stereoisomers we cannot exclude the possibility that *rac*-GR24 may interact with KAI2 thus interfering KAR signal transduction (Scaffidi et al., 2014) and consequently influencing root development (Villaécija-Aguilar et al., 2019). In Col-0 roots, TIS108 decreased the number of both staged-LRs, but in *35S:FLAG-GSNOR1* it increased the number of LR primordia. Based on these we can assume that in case of normal GSNOR level reduced SL level inhibits LR initiation, while in the presence of increased GSNOR activity or nitrate-derived NO SL inhibition leads to the induction of LR initiation. These signal interactions may be complex and the knowledge of other contributing factors would be necessary to fully explain the observed effects. It can be a concern that the effect of the analog and the inhibitor is not always the opposite. At the same time, it is conceivable that an optimal SL level is needed for normal root growth. Increasing (by the addition of GR24) or lowering (by the addition of TIS108) the optimal SL level may result in similarly inhibited growth processes.

**Figure 6:**
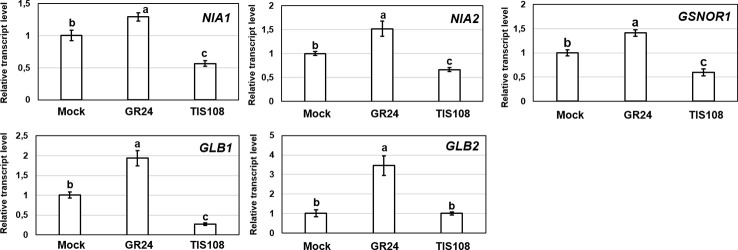
Relative transcript level of selected NO-associated genes (*NIA1, NIA2, GSNOR1, GLB1, GLB2*) in Col-0 *Arabidopsis* grown under without (Mock) or with GR24 (2 µM) or TIS108 (5 µM). Different letters indicate significant differences according to Duncan's test (*n =* 3, P ≤ 0.05). Data were normalized using the *A. thaliana ACTIN2* and *GAPDH2* genes as internal controls. The relative transcript level in Col-0 control samples was arbitrarily considered to be 1 for each gene.

Treatment with GR24 resulted in significantly increased NO content in *Arabidopsis* roots (Kolbert, 2019). As for NO-associated genes, the expressions of *NIA1* and *NIA2* as well as *GSNOR1* didn't show any relevant modification in the presence of GR24 (**Figure 6**). In contrast, nitrogen regulatory protein P-II homolog (*GLB1*) and non-symbiotic hemoglobin 2 (*GLB2*) genes were upregulated by GR24. The *GLB* genes encode plant hemoglobins which may act as NO scavengers (Hebelstrup and Jensen, 2008; Hebelstrup et al., 2012; Mira et al., 2015). In this experimental system; however, *GLB1* and *GLB2* upregulation induced by GR24 did not lead to NO scavenging, but instead GR24 induced NO production (Kolbert, 2019). This seems to be an interesting contradiction that needs further research.

### The Effect of NO Donor and Scavenger on SL-Associated Genes and Root System of *Arabidopsis*

**Figure 7:**
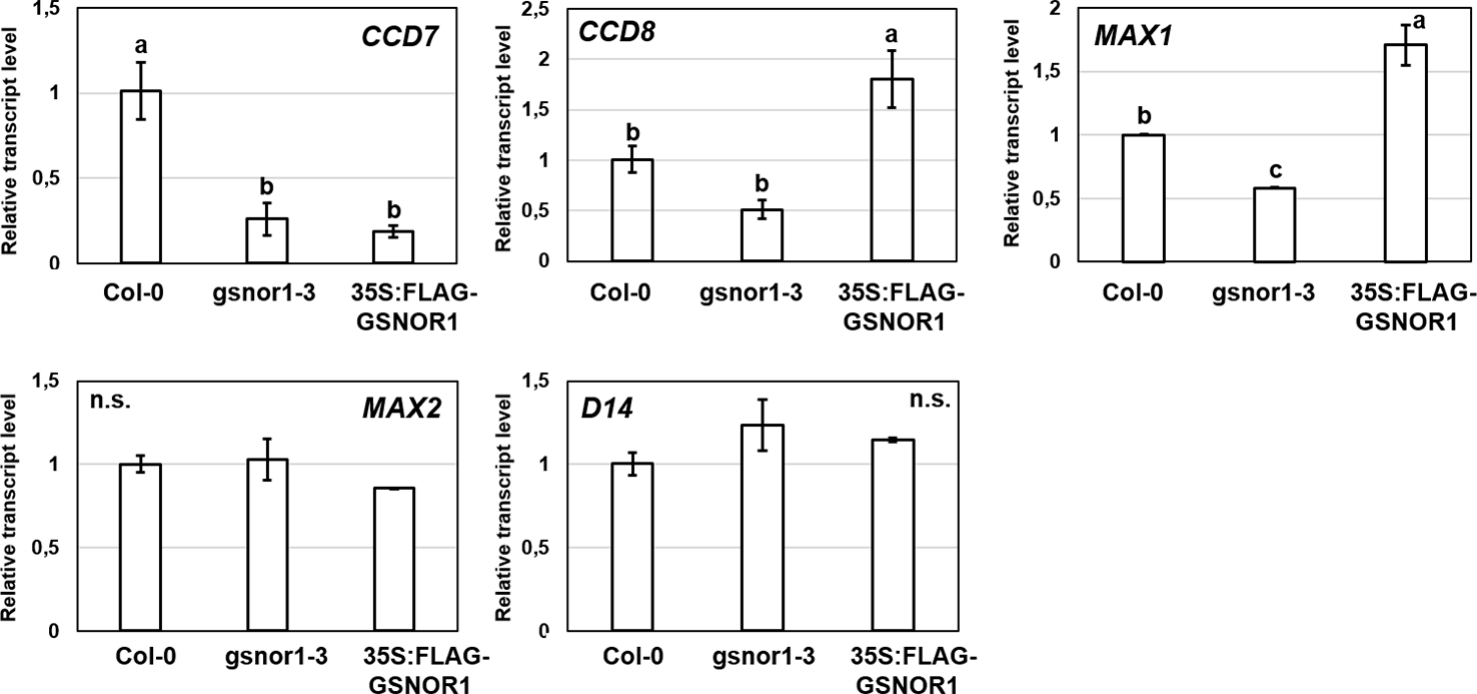
Relative transcript level of selected SL-associated genes in Col-0, *gsnor1-3* and *35S:FLAG-GSNOR1 Arabidopsis* seedlings grown during stress-free conditions. Different letters indicate significant differences according to Duncan's test (*n =* 3, P ≤ 0.05). Data were normalized using the *A. thaliana ACTIN2* and *GAPDH2* genes as internal controls. The relative transcript level in Col-0 control samples was arbitrarily considered to be 1 for each gene.

**Figure 8:**
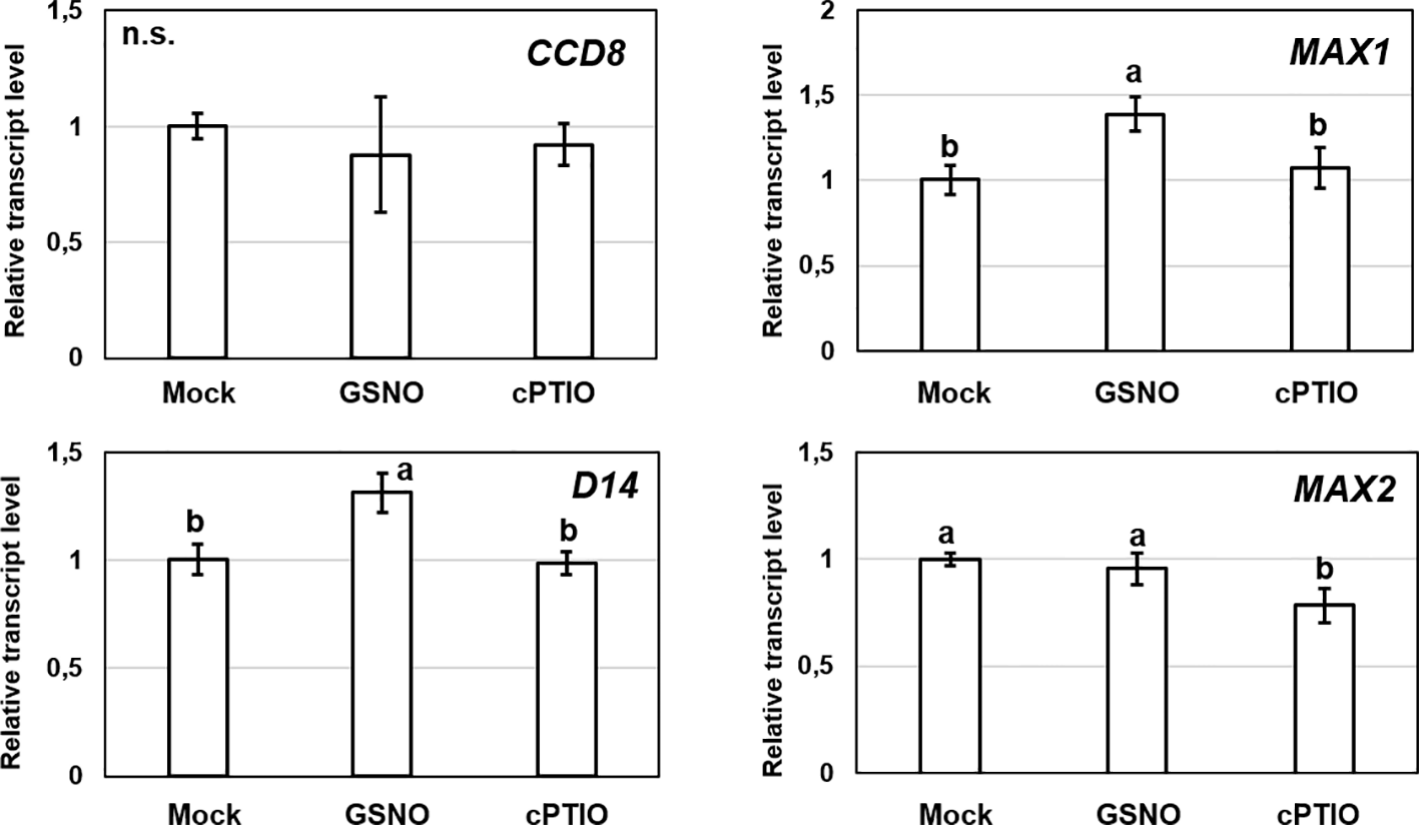
Relative transcript level of selected SL-associated genes (*CCD7, CCD8, MAX1, MAX2, D14*) in Col-0 *Arabidopsis* grown in the absence (Mock) or in the presence of GSNO (250 µM) or cPTIO (800 µM). Different letters indicate significant differences according to Duncan's test (*n =* 3, P ≤ 0.05). Data were normalized using the *A. thaliana ACTIN2* and *GAPDH2* genes as internal controls. The relative transcript level in Col-0 control samples was arbitrarily considered to be 1 for each gene.

We were interested also in reverse interplay, *i.e.*, whether under- or overproduction of GSNOR enzyme affects the expression of SL-associated genes (**Figure 7**). The examined genes (*CCD7, CCD8, MAX1*) involved in the synthesis of SLs showed down-regulation in GSNOR-deficient *Arabidopsis* compared to Col-0. This indicates that in case of low GSNOR activity, SL biosynthesis is inhibited. This further supports the interaction between GSNO metabolism and SL production in *Arabidopsis*. In addition, *CCD7* was down-regulated also in GSNOR overproducing *35S:FLAG-GSNOR1* seedlings. In contrast, the expressions of SL signaling genes (*D14* and *MAX2*) were not altered by GSNOR deficiency or overproduction. However, this was not supported by pharmacological treatments (GSNO or cPTIO), because we didn't observe relevant up- or downregulation of SL-associated genes (*CCD7, CCD8, MAX1, MAX2, D14*) in the presence of NO donor (GSNO) or scavenger (cPTIO) treatments (**Figure 8**). However, Castillo et al. (2018) observed larger induction in the expression of MAX1 and MAX2 in *Arabidopsis* seedlings due to NO treatment. From the applied 250 µM GSNO solution approx. 220 nM NO liberated over 15 min during the same circumstances as the plant treatments took place (**Figure S2**).

**Figure 9:**
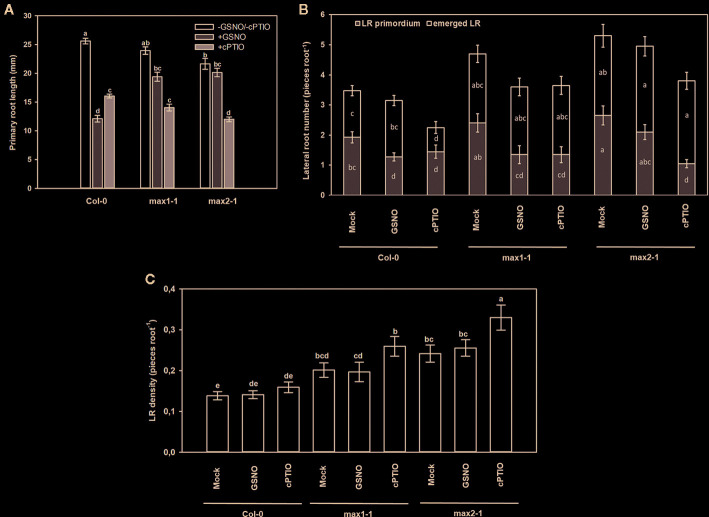
Primary root length (mm, **A**), lateral root number (number root^−1^, **B**) and lateral root density (number mm^−1^, **C**) in Col-0, *max1-1, max2-1 Arabidopsis* seedlings grown in the absence (Mock) or in the presence of GSNO (250 µM) or cPTIO (800 µM) for 3 days. Different letters indicate significant differences according to Duncan's test (*n =* 20, P ≤ 0.05).

To further investigate this interaction, GSNO and cPTIO treatments were applied, and the responses of *max* mutants were examined (**Figure 9**). Exogenous GSNO treatment resulted in 50% root shortening in Col-0, whereas this effect was absent in *max* mutants suggesting that the examined SL (and KAR) mutants are GSNO-insensitive and that SLs (and/or possibly KAR) are needed for GSNO-induced root shortening. Similar results were obtained in *Arabidopsis* hypocotyls, where NO-triggered shortening was not observed in *max1*, *max2* and *max4* mutants (Castillo et al., 2018). According to Fernández-Marcos et al. (2011) GSNO inhibits root meristem activity through the reduction of PIN1-dependent auxin transport. Since SLs were proved to negatively regulate PIN proteins in *Arabidopsis* roots (Ruyter-Spira et al., 2011), we can assume that GSNO may exert its effect on PINs *via* inducing SL (and/or possibly KAR) synthesis and/or signaling; although the link between NO, PINs and SL (and KAR) should be clarified by future research. The NO scavenger cPTIO shortened PRs to a similar extent in all three plant lines (*Col-0, max1-1, max2-1*). Moreover, GSNO inhibited LR initiation and slightly increased LR emergence of Col-0, while cPTIO supplementation decreased the number of both types of LR. In *max1-1* and *max2-1* seedlings, LR emergence seemed to be insensitive to NO donor or scavenger. However, GSNO treatment caused reduction in the number of LR primordia of the *max1-1* mutant, and cPTIO treatment decreased LR initiation in both *max* mutants. Just like the matching effects of SL analog and inhibitor, the effects of NO donor and scavenger proved also to be often similar to each other, indicating the necessity of an optimal NO level for optimal root development.

## Conclusion

The majority of the articles dealing with SL–NO interplay uses pharmacological approach and focuses on the root system of crops grown with special nutrient supply (excess nitrate or nitrogen- or phosphor deficiency). This study combines molecular biological and pharmacological approaches in order to reveal interactions between NO and SLs as growth regulating signals in the model plant *Arabidopsis thaliana* grown in stress-free conditions. As this study used *max2-1* mutant and rac-GR24, the observed effects might be non-specific to SL signaling, and the involvement of KAR signal pathway in this system cannot be ruled out. We observed for the first time that SL (and/or KAR)-deficiency resulted in elevated NO and SNO levels due to decreased GSNOR protein abundance and activity indicating that there is a signal interaction between SLs (and/or KAR) and GSNOR-regulated levels of NO/SNO. This was further supported by the down-regulation of SL biosynthetic genes (*CCD7, CCD8* and *MAX1*) in *gsnor1-3* containing elevated NO/SNO levels. Based on the more pronounced sensitivity of *gsnor1-3* to GR24, we suspected that functional GSNOR is needed to control NO/SNO levels during SL (and/or KAR)-induced PR elongation. Furthermore, SLs (and/or KAR) may be involved in GSNO-regulated PR shortening as suggested by the relative insensitivity of *max1-1* and *max2-1* mutants to exogenous GSNO. Collectively, our results indicate for the first time a connection between SL (and/or KAR) and GSNOR-regulated NO/SNO signals in *Arabidopsis thaliana* roots. Future studies should reveal the SL- or KAR-specificity of interactions with NO using *d14* and *kai2* mutants and GR24 stereoisomers. In the future, the possible involvement of auxin signaling as a common interacting factor of NO and SL during root development should also be examined. Additional research efforts should focus on the possible role of NO-dependent posttranslational modifications (S-nitrosation, tyrosine nitration) in relation to SL-regulated plant development.

## Data Availability Statement

The datasets generated for this study are available on request to the corresponding author.

## Author Contributions

DO performed the experiments and wrote the manuscript draft. GF performed the experiments and reviewed the manuscript. ÁM performed the experiments. AÖ performed experiments and reviewed the manuscript. ZK conceptualized the research, designed and directed the project, reviewed the manuscript draft, and wrote the final manuscript.

## Funding

This work was financed by the National Research, Development and Innovation Fund [Grant no. NKFI-6, K120383]. ZK was supported by the János Bolyai Research Scholarship of the Hungarian Academy of Sciences [Grant no. BO/00751/16/8]. DO was supported by UNKP-19-3-SZTE-201 New National Excellence Program of the Ministry for Innovation and Technology. Some of the experiments were carried out by ZK during a 3-month-long visit at the Institute of Biochemical Plant Pathology, Helmholtz Zentrum München supported by TEMPUS Foundation in the frame of the Hungarian Eötvös Scholarship (MAEÖ-1060-4/2017).

## Conflict of Interest

The authors declare that the research was conducted in the absence of any commercial or financial relationships that could be construed as a potential conflict of interest.
