# Supplementary material for: Strigolactones Interact With Nitric Oxide in Regulating Root System Architecture of Arabidopsis thaliana
Source: Front Plant Sci. 2020 Jul 3;11:1019. doi: 10.3389/fpls.2020.01019 (PMC7350899; doi:10.3389/fpls.2020.01019)
Supplement: Supplementary file 1 [file Table_1.docx]

| Gene name | Locus identifier | Forward primer (5’ to 3’) | Reverse primer (5’ to 3’) |
| --- | --- | --- | --- |
| ACTIN2 * | At3g18780 | GGTAACATTGTGCTCAGTGGTGG | AACGACCTTAATCTTCATGCTGC |
| GAPDH2 * | At1g13440 | AATGGAAAATTGACCGGAATGT | CGGTGAGATCAACAACTGAGACA |
| NR1 | At1g77760 | ACAAAGGCAAAGGCAACTTC | CCACATACATCTCGGTTTCGT |
| NR2 | At1g37130 | GCGTGGTGTCCCTCTCTG | TGATGCTCGTTCCGTATTTG |
| GSNOR1 | At5g43940 | ACTGATGGCGGTGTTGACTA | TTGGAACGGACGAGTTGATA |
| GLB1 | At2g16060 | AGCCTCACGCAATGTCTGTT | TTTCCCTGTTTTCCTCAGTTG |
| GLB2 | At3g10520 | TGAAGTCCCTCACAACAATCC | TCAGCCACTACCACCTTTCC |
| CCD7 | At2g44990 | CCTCTAAACGGGTGGAACAA | CGAATGGAAAATGGGGAAG |
| CCD8 | At4g32810 | TTGTCTTGTGCCCTCTTTCC | CTCATCATTGCTTTGGTTGTG |
| D14 | At3g03990 | GGTTTCTCAACGACGAGGAT | AACAGCAAGCGGAGCAAAT |
| MAX1 | At2g26170 | CGGGAAGAAACCAATCAAAG | TCGGAATCAGTAAGCCTAAGATG |
| MAX2 | At2g42620 | TGTGGTGGTTTCCTTGAGTCT | TTTGTATCCCTCGGTGAACG |

**Table S1** Primers used in this study. (*Papdi et al. 2008)
