# Supplementary figures and images for: Strigolactones Interact With Nitric Oxide in Regulating Root System Architecture of Arabidopsis thaliana
Source: Front Plant Sci. 2020 Jul 3;11:1019. doi: 10.3389/fpls.2020.01019 (PMC7350899; doi:10.3389/fpls.2020.01019)

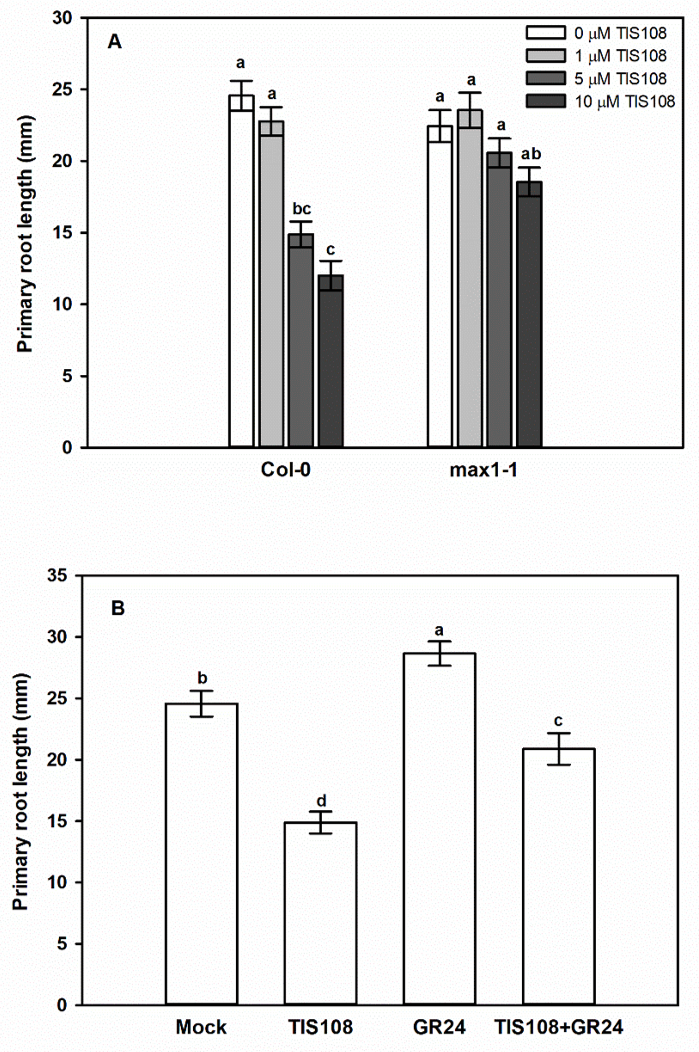

Supplement: Supplementary file 2 [file Image_1.tif]

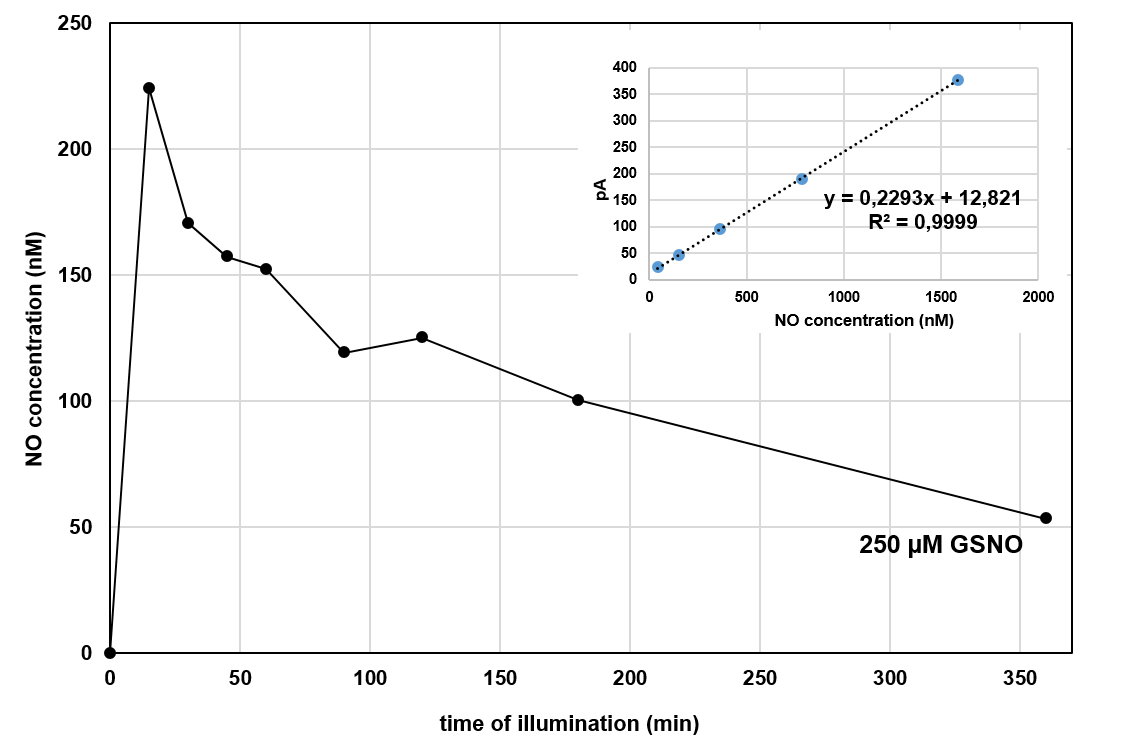

Supplement: Supplementary file 3 [file Image_2.tif]
